# Face/Off: The Interchangeable Side of *Candida Albicans*

**DOI:** 10.3389/fcimb.2019.00471

**Published:** 2020-01-28

**Authors:** Fabien Cottier, Rebecca A. Hall

**Affiliations:** ^1^School of Biosciences, Institute of Microbiology and Infection, University of Birmingham, Birmingham, United Kingdom; ^2^Kent Fungal Group, School of Biosciences, University of Kent, Canterbury, United Kingdom

**Keywords:** Candida, cell wall, innate immunity, morphogenesis, cell wall remodeling

## Abstract

Due to limited mobility, fungi, like most unicellular organisms, have evolved mechanisms to adapt to sudden chemical and/or physical variation in their environment. *Candida albicans* is recognized as a model organism to study eukaryotic responses to environmental changes, as this human commensal yeast but also opportunistic pathogen responds to numerous environmental cues through switching morphologies from yeast to hyphae growth. This mechanism is largely controlled by two major pathways: cAMP-PKA and MAPK, but each environmental signal is sensed by specific sensors. However, morphological switching is not the only response *C. albicans* exerts in response to environmental cues. Recently, fungal cell wall remodeling in response to host-derived environmental cues has been identified as a way for *C. albicans* to manipulate the innate immune system. The fungal cell wall is composed of a chitin skeleton linked to a network of β-glucan, which anchors proteins and mannans to the fungal cell surface. As localized on the cell surface, these molecules drive interactions with the environment and other cells, particularly with host immune cells. *C. albicans* is recognized by immune cells such as neutrophils and macrophages via pathogen recognition receptors (PRRs) that bind different components of the cell wall. While β-glucan and mannan are proinflammatory molecules, chitin can induce anti-inflammatory responses. Interestingly, *C. albicans* is able to regulate the exposure of these pathogen-associated molecular patterns (PAMPs) according to environmental cues resulting in a modulation of the host immune response. This review describes the mechanisms involved in *C. albicans* response to environmental changes and their effect on immune recognition.

## Introduction

*Candida albicans* is an opportunistic fungal pathogen of man and is associated with infections ranging from superficial mucosal infections to life-threatening disseminated disease. In 1853, Charles Philippe Robin drew one of the first representations of *C. albicans*, from a muguet infection isolate. On his illustration, yeasts, filaments, and clusters of blastoconidida were represented (Histoire Naturelle des Végétaux Parasites qui Croissent sur l'Homme et sur les Animaux Vivants.). However, since then, *C. albicans* has been described to exist in eight different morphotypes including four yeast morphologies (white, opaque, gray, and gut), two hyphal morphologies (linear and sinusoidal), pseudo-hyphae, and chlamydospores (Hayes, 1966; Slutsky et al., 1987; Brand et al., 2009; Pande et al., 2013; Tao et al., 2014). In addition to this large diversity in shape, *C. albicans* can also exist in unicellular culture, biofilms, and micro-colonies (McCall et al., 2018). This polymorphic nature of *C. albicans* is a great example of a protean organism.

Most of these various morphotypes have been observed clinically, suggesting that each morphotype plays a specialized role in infection. On one hand, *C. albicans* is a commensal yeast, frequently isolated from organs such as the female reproductive tract and the gastrointestinal tract where it can represent up to 0.1% of the microbiome (Beigi et al., 2004; Cottier et al., 2018). However, *C. albicans* is also responsible for mild skin or mucosal infections such as the muguet (oral candidiasis) or vulvovaginal candidiasis, which affects 70% of women (Jeanmonod and Jeanmonod, 2019). On the other end of the spectrum, *C. albicans* bloodstream infections represent over 50% of all reported candidemias and affects eight out of 100,000 people causing life-threatening infections in immuno-deficient patients (Pfaller and Diekema, 2007). The breadth and depth of candidiasis bring pressure on medical services as time and cost of treatment amount to several billions dollars per year (Moran et al., 2010).

Historically, the morphological yeast-to-hyphae switch of *C. albicans* has been an intense focus of research as it is one of the main drivers of fungal virulence, and is a visually drastic phenotype. Cells locked in either of these two forms display a lower virulence in murine infection models (Lo et al., 1997; Saville et al., 2003). However, the discovery of the novel hyphal specific fungal cytolytic toxin, candidalysin, revealed that it is the production of this toxin that is essential for virulence, rather than virulence being truly attributed to hyphal formation alone, as *ece1* deficient hyphae are attenuated in virulence (Moyes et al., 2016). Additional work enhanced this dichotomy between yeast-to-hyphae switching and pathogenicity (Noble et al., 2010). Now, it is appreciated that it is not only the morphology of *C. albicans* that is important for virulence, but also the metabolic state of the fungus. Indeed, while hyphae and proteins such as extracellular hydrolytic enzymes and candidalysin could be considered as the spear of *C. albicans*, cell wall remodeling could be viewed as a shield against the innate immune response. This review explores the environmental factors influencing morphological switches in *C. albicans* but also most recent studies demonstrating the role of the environment in regulating the composition of the fungal cell wall. Finally, how this plasticity of *C. albicans* aids exploitation of the innate immune system will be explored.

### *C. albicans* Morphology

#### Opaque, GUT, and Gray Cells

**Figure 1:**
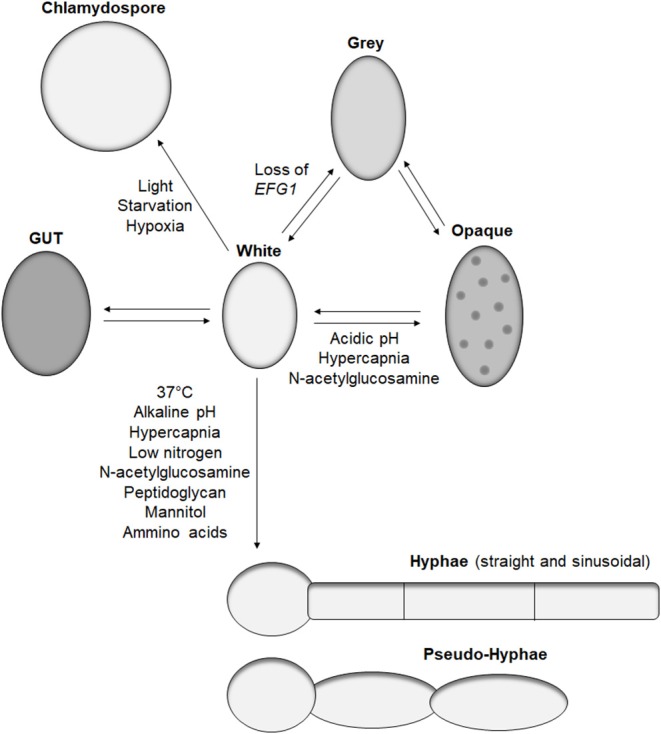
*Candida albicans* morphologies. Representation of the reported morphotypes of *C. albicans* and the known cues triggering morphological switches.

The main morphotype of *C. albicans* is a yeast shape known as white cells. These cells are common in *in vitro* studies, have a round-to-oval shape, and measure around 5–6 μm (Klis et al., 2014). This form is widely observed in *in vivo* studies and clinical isolates and is efficient for dissemination and adhesion to surfaces. The first morphotype derived from the white cell was the opaque cell (Figure 1), the mating competent form of *C. albicans* (Slutsky et al., 1987). Opaque cells have an ellipsoidal shape of around 7 μm long, with pimples on the surface (Lockhart et al., 2002). These cells are the result of a loss of heterozygosity at the *MTL* locus, leading to the formation of **a** and **α** cells (Miller and Johnson, 2002). Sexually competent *MTL*α cells produce a pheromone that triggers “shmooing,” the formation of a conjugation tube, in *MTL*a opaque cells (Lockhart et al., 2003). This initial mating steps ends with the fusion of the two nuclei and formation of a tetraploid cell. Several environmental factors induce white-to-opaque switching like acidic pH, hypercapnia, and exposure to N-acetylglucosamine (Huang et al., 2009, 2010; Sun et al., 2015). These cues are sensed by two systems, Sac7/Rho1 and Ras1, ending in the regulation of the master white to opaque transcriptional regulator Wor1 (Yang et al., 2018).

Recently, the white-to-opaque switch was reported to be part of a tristable phenotypic switch with the discovery of gray cells (Tao et al., 2014). These cells have also an ellipsoidal shape and are similar in length to the opaque cell but with a smaller diameter and do not have pimples on their surface. While gray cells are thousand-fold more efficient than white cells at mating, gray cells are hundred-fold less efficient than opaque cells at mating (Tao et al., 2014). Similar to opaque cells, gray cells are the result of a genetic alteration. Earlier this year, Liang et al. demonstrated that under certain conditions, such as passage through the mammalian gastrointestinal tract, *C. albicans* can gain mutations in the transcription factor Efg1, primarily known for its role in morphogenesis, but also promotes the formation of white cells (Liang et al., 2019). A strain of *C. albicans* hemizygous for *EFG1* has a white cell morphology, but any functional loss of the remaining *EFG1* wild-type allele results in formation of gray cells. Such events occur in the human host, with gray cells containing mutations in *EFG1* being isolated from clinical samples (Liang et al., 2019). Furthermore, gray cells display a fitness advantage in mice gastrointestinal colonization models, which correlates with previous work demonstrating the competitive advantage of an *efg1* mutant over a wild-type strain (Pierce and Kumamoto, 2012). However, these observations were performed in antibiotic-treated mice; therefore, the impact of *efg1* inactivation and gray cells in a complex gut microbiome remains to be characterized.

In addition to gray cells, another *C. albicans* morphotype, gastrointestinal induced transition (GUT), has been associated with gastrointestinal colonization. GUT cells are similar in size and shape to opaque cells but do not possess pimples on their surface (Pande et al., 2013). GUT cells are very similar to gray cells except GUT cells tend to be wider. GUT cells were identified during gastrointestinal colonization studies in mice using a strain over-expressing the transcription factor *WOR1*. Similarly to gray cells, GUT cells present a fitness advantage in gut colonization compared to control white cells (Pande et al., 2013). They are also significantly less efficient at mating than gray cells. The main difference between gray and GUT cells is a lack of stability, with GUT cells transitioning back to white cells outside the gut environment, indicating the requirement for an external stimulus to maintain this phenotype (Pande et al., 2013). Given the similarity between gray and GUT cells, it would be interesting to identify if GUT cells are the result of *EFG1* suppression, which is relieved when removed from the gut environment.

#### Yeast-to-Hyphal Transition

The most studied morphological switch of *C. albicans* is the formation of hyphae. During hyphal formation, yeast stop forming buds, and grow tube-shaped cells linked to each other by a septum. This structure allows communication between the cytoplasm of the interconnecting cells and creates a mycelium. Hyphae have a lower diameter compared to yeast, around 2.5–3.5 μm (Klis et al., 2014), but their length can easily be over 100 μm, surpassing the size of immune cells. This morphological transition is triggered by various known environmental cues present in the human host including 37°C, pH, hypercapnia, low nitrogen, N-acetylglucosamine, peptidoglycan, mannitol, or amino acids (Figure 1) (Noble et al., 2017). Most of these signals activate either the cAMP-PKA or MAPK pathways resulting in the activation of the transcription factors Efg1 and Cph1, respectively, as reviewed in Noble et al. (2017). Interestingly, deletion of the transcriptional factor *UME6*, which play a central role in morphogenesis of *C. albicans* (Carlisle and Kadosh, 2010), is deficient in hyphal formation in *in vitro* assays but is still able to produce hyphae in the mouse gastrointestinal tract, revealing the existence of at least one unidentified hyphal inducing signal (Witchley et al., 2019).

Hyphae are a major component of *C. albicans* biofilms, which have a higher resistance to antifungal drugs than planktonic cultures (Mathe and Van Dijck, 2013). However, hyphal differentiation is also a strategy for yeast to escape immune cells after phagocytosis. Initially, this escape mechanism was thought to be due to mechanical damage of the phagocyte. However, it is now understood that escape of *C. albicans* from macrophages is due to an intricate mechanism requiring several key processes. For example, hyphae can trigger phagocyte killing through caspase-1-dependent pyroptosis (Uwamahoro et al., 2014; Wellington et al., 2014), and then once extracellular, these hyphae induce glucose starvation resulting in enhanced macrophage cell death (Tucey et al., 2018). Alternatively, hyphal differentiation in immune cells induces the production of the cytotoxin candidalysin resulting in phagocyte membrane damage (Kasper et al., 2018).

In addition to these straight tubular hyphae, sinusoidal or helical hyphal growth has been reported in *C. albicans* when cultivated on surfaces (Sherwood-Higham et al., 1994; Brand et al., 2009; McCall et al., 2018), and are associated with antifungal resistance (Sherwood-Higham et al., 1994). However, hyphae are not solely a morphological switch, but they are also associated with important transcriptional regulation of virulence factors including secreted aspartyl protease, adhesins and superoxide dismutase (Mayer et al., 2013), and, more recently, the cytotoxic peptide candidalysin encoded by *ECE1* (Moyes et al., 2016).

Pseudo-hyphae are a morphological form sharing aspects of yeast, as cells have an oval to ellipsoid shape, and like hyphae can form a mycelium. Contrary to hyphae, pseudo-hyphae are not tube shaped but are elongated yeasts with separation between each cell that is easily identified by a change in diameter (Sudbery et al., 2004). Pseudo-hyphae are observed in response to various cues inducing hyphae formation, but none are known to differentiate solely pseudo-hyphae. The relevance of this morphotype in *C. albicans* life cycle remains to be studied.

#### Chlamydospores

The last identified *C. albicans* morphotype is the chlamydospore. These large round cells have a diameter of 7–8 μm and are characterized by a thick cell wall (Hayes, 1966). However, the latter does not provide significant resistance to heat or starvation (Citiulo et al., 2009). Nevertheless, several environmental cues have been identified to trigger chlamydospore differentiation such as light, starvation, or hypoxia, but the biological function of this morphotype remains to be elucidated (Bottcher et al., 2016).

As reported, *C. albicans* responds to various environmental cues by producing different morphotypes. While cell shape is immediately observable, each of these forms is also associated with major transcriptional responses ranging from virulence factors to metabolic pathway regulation (Tuch et al., 2010; Palige et al., 2013; Guedouari et al., 2014; Tao et al., 2014; Grahl et al., 2015; Koch et al., 2018). Several environmental signals have also been shown to influence the expression of secreted, as well as cell wall-associated, proteins. As a result, cell wall remodeling has recently been described to occur in response to these changes (Ballou et al., 2016; Sherrington et al., 2017; Lopes et al., 2018). For example, chitin, which is a polymer of β-(1,4)-*N*-acetylglucosamine, is regulated by a balance between production via chitin synthases (*CHS1-3, CHS8*) and degradation by chitinases (*CHT1-4*), allowing *C. albicans* to reshape its chitin network when needed (Selvaggini et al., 2004). Cell wall characterization and versatility is another aspect of *C. albicans* ever-changing faces whose understanding is critical as cell wall is the initial point of contact between fungal cells and the host immune system (Hopke et al., 2018).

### *C. albicans* Cell Wall Remodeling

*C. albicans* asymptomatically colonize human skin, vagina, and gastrointestinal tract, niches where temperature, gas composition, or carbon source vary greatly through space as well as time (Grahl et al., 2012). As discussed above, *C. albicans* responds to changes in these environmental cues by altering its shape. Transcriptional responses of *C. albicans* to these signals have been characterized and point toward customized profiles of gene expression. Interestingly, genes involved in cell wall structure have been shown to be regulated in response to pH, morphogenesis, hypoxia, or carbon source (Bensen et al., 2004; Synnott et al., 2010; Ballou et al., 2016).

The *C. albicans* cell wall is composed of three major components: (i) a chitin skeleton, (ii) a network of β-(1,3)- and β-(1,6)-glucan, and (iii) highly glycosylated proteins sometimes referred to as mannoproteins. In *C. albicans* white cells, chitin represents 1–2% of cell wall dry weight, while β-1,6-glucan and β-1,3-glucan amount for, respectively, 20 and 40% and the remaining 35–40% correspond to the mannoproteins (Navarro-Arias et al., 2019). With the help of transmission electron microscopy, we can now visualize the individual layers of the cell wall. The inner layer (chitin and glucan) forms a relatively electron dense region, while the outer layer formed by the mannoproteins is composed of densely packed fibrils extending away from the fungus (Chaffin et al., 1998). Adaptation to environmental conditions alters the composition of both cell wall layers. For example, the thickness of the outer mannan layer in white cells is dependent on temperature and nutrient availability, with cells grown in rich media at 37°C displaying shorter mannan fibrils than cells grown in minimal media at 30°C (Ene et al., 2012; Sherrington et al., 2017). Temperature also regulates the thickness of the inner cell wall layer, again with higher temperatures reducing the overall thickness of the glucan–chitin layer. The thickness of the hyphal cell wall is comparable to the yeast cell wall, but can be greater at the hyphal tip (Knafler et al., 2019). The thickness of the cell wall is also modulated by pH, with growth in acidic media resulting in a 50% reduction in the outer mannan layer, while the inner layer is not significantly affected by pH (Sherrington et al., 2017). Interestingly, alkaline pH has the opposite effect, increasing the outer mannan layer. A change in carbon source, from glucose to lactate in minimum media, or exposure to hypoxic conditions also reduces the thickness of the inner layer by 50% (Ballou et al., 2016).

#### Cell Wall Remodeling in Response to Lactate

Despite the fact that the inner layer is covered by the outer layer of mannoproteins, which has previously been described as a molecular shield to protect the highly immunogenic glucan from the innate immune system, adaptation to environmental signals results in exposure of the glucan and chitin network, making it accessible to immune cells (Sherrington et al., 2017). Notably, β-(1,3)-glucans are recognized by the pattern recognition receptor (PRR) Dectin-1 triggering phagocytosis of *C. albicans* by neutrophils and macrophages (Taylor et al., 2007). Cell wall remodeling, resulting in the masking and unmasking of PAMPs like β-glucan and chitin, is an intricate mechanism of *C. albicans* survival and virulence in the human body. Similar to cell shape or cell wall thickness, PAMP exposure is influenced by environmental cues like carbon sources. Indeed, cells incubated for 4 h at 30°C in minimal media supplemented with lactate display masking of the β-glucan (>50% reduction in glucan exposure) (Ballou et al., 2016). This decline in β-glucan exposure resulted in lower phagocytic recruitment (Ene et al., 2013). Cell wall remodeling in response to lactate involves the plasma-membrane receptor Gpr1 and its associated Gα protein Gpa2 as a double mutant exhibited a lack of glucan masking in the presence of lactate. Interestingly, these two proteins are known to activate the cAMP-PKA pathway during the yeast-to-hyphal transition, but mutants in this pathway (*cyr1*Δ and *pde2*Δ) did not have any impact on cell wall remodeling in response to lactate. Mutants involved in the calcium signaling pathway (*cna1* and *cnb1*) were also unaffected for lactate-induced cell wall remodeling, while the downstream regulator Crz1 was shown to be essential for β-glucan masking (Ballou et al., 2016). These experiments suggest the existence of a novel Crz1-dependent signaling pathway involved in cell wall remodeling in response to lactate.

#### Cell Wall Remodeling in Response to Hypoxia

A second environmental factor inducing a 2-fold decrease in glucan exposure is hypoxia, a cue particularly important as *C. albicans* encounters low oxygen environments during colonization of internal organs (Pradhan et al., 2018). However, none of the components previously identified for lactate-induced glucan masking play a role in the response to hypoxia. Instead, hypoxia-induced cell wall remodeling is regulated predominately via the cAMP-PKA pathway (Pradhan et al., 2018). Here, hypoxia triggers the production of reactive oxygen species (ROS) from the mitochondria in a Goa1- and Upc2-dependent mechanism. This ROS is then converted to hydrogen peroxide by Sod1, which then activates the cAMP-PKA signaling cascade, through an as yet to be identified mechanism, which then functions to induce glucan masking (Pradhan et al., 2018). Interestingly, inhibition of mitochondrial respiration by exposure to sodium nitroprusside (SNP) and alicyl-hydroxamic acid (SHAM) for 18 h in rich media at 30 or 37°C induces both β-glucan and chitin exposure (Duvenage et al., 2019). The role of the cAMP-PKA pathway in this condition has not been investigated, but contrary to hypoxic exposure, *UPC2* inactivation did not have any impact on PAMP exposure. However, the transcription factor Sko1, involved in the cell wall damage response, displayed an increase in chitin exposure when incubated with SNP and SHAM (Duvenage et al., 2019), suggesting that multiple mitochondrial inputs regulate cell wall remodeling. Importantly, *C. albicans* growth and viability are significantly reduced when exposed to these molecules, which could have secondary effects on PAMP exposure.

#### Cell Wall Remodeling in Response to Environmental pH

Environmental pH also induces cell wall remodeling, but in contrast to lactate and hypoxia, acidic environments promote the exposure of glucan and chitin exposure (Sherrington et al., 2017). Crz1 was shown to have no role in chitin exposure in response to acidic environment, but the transcription factors Rim101 and, to a lesser extent, Bcr1 play key roles in regulating chitin exposure. This effect was mediated through the regulation of the chitinase, *CHT2*, suggesting that this enzyme is responsible for the unmasking of chitin in the cell wall. Inactivation of *CHT2* induced a significant increase in chitin exposure at pH 6 compared to a control strain (Sherrington et al., 2017). Furthermore, *CHT2* transcript levels are up-regulated during growth at pH 6 compared to pH 4, but this regulation is absent in both *rim101*Δ and *bcr1*Δ mutants (Sherrington et al., 2017). Noticeably, *CHT2* transcript levels have been reported to be a target of She3, an mRNA transporter to the cell wall, and inactivation of *SHE3* resulted in an increase of chitin exposure at pH 6 similar to a *cht2* mutant (Sherrington et al., 2017). Altogether, acidic environments inactivate the Rim101 and Bcr1 pathways, reducing *CHT2* expression, leading to a lack of chitinase in the cell wall and enhancing exposure of chitin in the cell wall (Sherrington et al., 2017).

#### Cell Wall Remodeling in Response to Multiple Signals

While these environmental cues are studied separately *in vitro*, in the host, *C. albicans* encounters many of these signals, and more, simultaneously. For example, in murine subdermal abscess models, infiltration of neutrophils to the site of *C. albicans* infection contributes to the formation of a hypoxic environment (Lopes et al., 2018). Furthermore, neutrophil-dependent production of lactate is increased by 40% in anoxia compared to normoxia *in vitro* and supernatants of such cultures reduce glucan exposure. Therefore, neutrophil recruitment to sites of *C. albicans* infection could form a local environment that is low on oxygen and rich in lactate (Lopes et al., 2018). This metabolic rewiring was confirmed in human monocytes, as glucose consumption and lactate production were increased in the supernatants of monocytes exposed to *C. albicans* compared to non-stimulated cells (Dominguez-Andres et al., 2017). *In vivo*, glycolysis was also shown to be up-regulated in patients with fungal sepsis compared to healthy controls during genome-wide transcriptional profiles of blood (Cheng et al., 2016). Such conditions would trigger cell wall remodeling, masking critical PAMPs such as glucan, preventing immune recognition, and allowing *C. albicans* colonization and evasion of the host.

**Figure 2:**
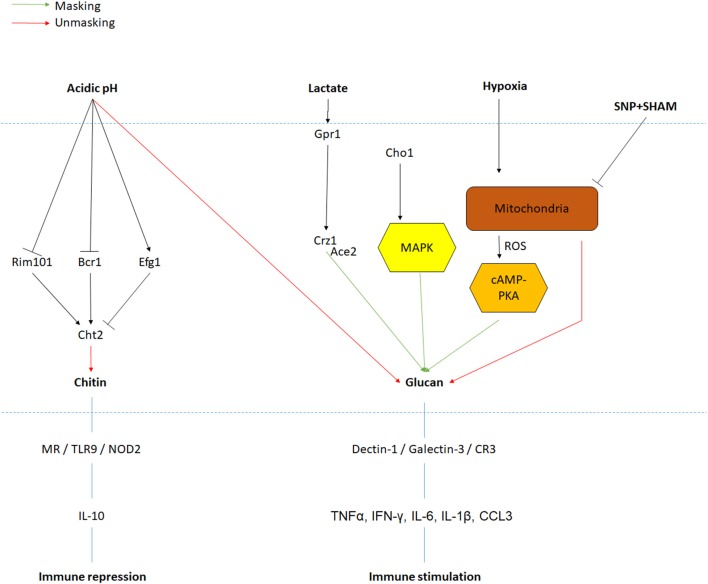
PAMPs exposure regulation in response to environmental signals. Signals and their associated pathway are indicated in black. Red and green arrows are, respectively, representing increases in PAMPs unmasking and masking. Major PRRs and immune response to PAMPs recognition are also represented.

Taken together, it appears as though *C. albicans* has evolved several signaling pathways that, in response to particular stimuli, regulate cell wall remodeling (Figure 2). Currently only a few host-derived environmental signals have been investigated for their ability to induce cell wall remodeling, and it is likely that this cell wall plasticity will extend to many other environmental parameters. Given that the cell wall forms the exterior of the fungus, and is in direct contact with the innate immune system, these cell wall perturbations will have a significant effect on how the invading pathogen is sensed by the innate immune cells.

### Immune Responses

Using cell wall remodeling, *C. albicans* is able to partially control the innate immune response mounted against it. Masking and unmasking of PAMPs such as β-glucan and chitin can trigger immune suppression as well as immune tolerance (Romani, 2011). Indeed, *C. albicans* is not alone in shielding its glucan to avoid detection form the innate immune system. Upon encountering the host environment, *Histoplasma capsulatum* completely removes its β-glucan from the cell wall, replacing it with the less proinflammatory α-glucan. Residual β-glucan is trimmed from the cell surface via the Endo and Exoglucanases Eng1 and Exg8 (Garfoot et al., 2017).

#### Recognition of Glucan

As mentioned previously, β-glucans are the main component of *C. albicans* cell wall as these β-D-glucose polysaccharides represent around 60% of the yeast dried cell wall mass (Ruiz-Herrera et al., 2006). β-(1,3)-glucans are known to be the ligand of the PRR Dectin-1. Interestingly, while live *C. albicans* yeast were able to activate Dectin-1, a similar amount of hyphal biomass did not (Gantner et al., 2005). This was attributed to the fact that budding yeasts have bud scars on their surface that expose glucan, which are lacking in hyphae. This mechanism demonstrates the relevance of PAMP exposure in the activation of the immune response. Indeed, the level of β-glucan exposure correlates with macrophage phagocytic rates, with environments that promote glucan exposure, also promoting phagocytosis (Ballou et al., 2016; Sherrington et al., 2017; Lopes et al., 2018). Similar observations were made when looking at the recruitment of innate immune cells to the site of infection, with glucan-exposed cells recruiting significantly more phagocytes than cells that had masked their glucan (Ballou et al., 2016; Sherrington et al., 2017). Additionally, human peripheral blood mononuclear cells (PBMCs) exposed to *C. albicans* grown under hypoxic conditions secrete a lower amount of IL-10 and RANTES and to a lesser extent TNFα and CCL3 (Pradhan et al., 2018). While for lactate-grown *C. albicans*, PBMCs are shown to produce a lower amount of IL-17, but human M1-activated monocyte-derived macrophages secreted a lower amount of TNFα and CCL3 compared to the glucose grown control (Ballou et al., 2016). As expected, an increase in β-glucan exposure such as in response to acidic environment induced the opposite secretion of cytokines. Indeed, *C. albicans* cells grown at pH 4 induce a higher level of TNFα, IFN-γ, IL-6, and IL-1β secretion by PBMCs compared to cells grown at pH 6 (Sherrington et al., 2017). Therefore, by controlling the level of glucan exposure, *C. albicans* can affect the level of proinflammatory cytokine produced and the recruitment of immune cells.

Given that Dectin-1 is the predominate β-glucan recognition receptor on innate immune cells, it is not surprising that the above effects are Dectin-1 dependent. However, although blocking Dectin-1 reduced phagocytosis of *C. albicans* with high glucan exposure, this had minimal effect on the ability of *C. albicans* to attach to the macrophage surface (Sherrington et al., 2017). Hence, Dectin-1 is involved in the enhancement of phagocytosis in response to β-glucans exposure but is not the primary receptor for the attachment of macrophages to *C. albicans*. Dectin-1 knockout mice were shown to be significantly more sensitive to *C. albicans* intravenous infection, as well as displaying a large fungal burden in the mouse gastrointestinal tract compared to the control group (Taylor et al., 2007). Furthermore, in an intraperitoneal infection model, Dectin-1 knockout mice were less able to recruit neutrophils, monocytes, and eosinophils to the site of infection than wild-type mice and this was associated with a decrease in IL-6, CCL2, CCL3, G-CSF, and GM-CSF production (Taylor et al., 2007). Interestingly, *C. albicans* virulence as well as kidney fungal burden in Dectin-1 knockout mice is dependent on the yeast strain (Marakalala et al., 2013). Indeed, *C. albicans* cells from clade 4 do not display increased virulence compared to wild-type mice. This variable influence of Dectin-1 in *C. albicans* recognition seems to be affected by cell wall chitin content (Marakalala et al., 2013).

While Dectin-1 is central to β-glucan recognition on various immune cells such as macrophage, neutrophils, or dendritic cells, other receptors have been identified to act in this process. Galectin-3, a soluble protein, physically interacts with Dectin-1 to enhance TNFα production in macrophages exposed to *C. albicans* β-glucan (Esteban et al., 2011). Galectin-3 also modulates complement receptor 3 (CR3) activation. CR3 is able to bind β-(1,6)-glucan, inducing phagocytosis and ROS production. However, galectin-3-deficient neutrophils had a higher level of ROS than the control strain after exposure to *C. albicans*. This effect is mediated via a physical interaction between galectin-3 with the CR3 downstream component Syk (Wu et al., 2017). Interestingly, additional receptors are suspected to recognize β-glucan as PBMCs have been identified to produce IL-1Ra in response to *C. albicans* hyphae in a Dectin-1- and CR3-independent manner (Smeekens et al., 2015).

#### Recognition of Chitin

As a component of the inner cell wall, chitin is normally not accessible to immune cells (Mora-Montes et al., 2011), but acidic environments increase chitin exposure in *C. albicans*. Purified chitin from *C. albicans* alters immune recognition of this yeast. Pre-treatment of PBMCs with chitin before exposure to live *C. albicans* decreases the secretion of proinflammatory cytokines, particularly IL-1β, IL-6, and TNFα (Mora-Montes et al., 2011). This effect is mediated by Dectin-1, as addition of chitin to Dectin-1-deficient mouse macrophages failed to inhibit the production of TNFα. However, this effect does not involve direct binding between chitin and Dectin-1, implying cooperation between multiple PRRs (Mora-Montes et al., 2011). In addition to dampening the secretion of proinflammatory cytokines, chitin alone is sufficient to induce the secretion of anti-inflammatory cytokines like IL-10 (Wagener et al., 2014). In conflicting studies, chitin also induced the secretion of proinflammatory cytokines including IL-6 and TNFα (Wagener et al., 2014). However, it appears as though the size of the chitin particle plays an interesting role here. While small chitin particles are anti-inflammatory and inhibit the secretion of proinflammatory cytokines, larger chitin particles (40–70 μm) function to induce proinflammatory cytokine production. These opposite effects are driven through the interaction with different PRRs. Smaller chitin particles appear to mediate their effects through Dectin-1/TLR2 signaling and the mannose receptor (MR) (Da Silva et al., 2009). *NOD2, TLR9*, and their downstream effectors are also important for chitin-induced secretion of IL-10 (Wagener et al., 2014), suggesting that a complex network is at play here. The anti-inflammatory response induced by chitin could be the result of co-evolution between the host and *C. albicans* preventing further immune inflammation once the yeast has been killed. However, *C. albicans* could take advantage of this mechanism in a certain environment or condition to lessen the immune response.

#### Recognition of Mannan

Finally, mannoproteins from *C. albicans* outer cell wall layer are themselves targets of PRRs, but their composition also affects the exposure of the underlying network of glucan. Using super resolution imagery of several mannosylation mutants (*mnn2*Δ, *mnn2*Δ/*mmn6*Δ, *mnn2*Δ/*mnn21*Δ/*mnn22*Δ/*mnn23*Δ/*mnn24*Δ/*mnn26*Δ), Graus et al. were able to demonstrate that a decrease in acid labile mannan abundance and α-mannan backbone length results in a larger region of glucan exposure on the yeast surface (Graus et al., 2018). By controlling mannosylation on surface proteins, *C. albicans* possess a mechanism to adjust the level of β-glucan being exposed. These results are in line with previous observations showing that inactivation of mannosyltransferase induces a higher level of glucan exposure and increases the level of TNFα secretion (Mora-Montes et al., 2010; Ueno et al., 2013; Zhang et al., 2016).

PRRs such as DC-SIGN recognize *N*-linked mannans and trigger *C. albicans* phagocytosis by human dendritic cells as well as the secretion of IL-6 (Cambi et al., 2008). The mannose receptor (MR) is a C-type lectin present on the surfaces of macrophages and is able to recognize mannose conformation. MR can be shed from the macrophage surface and be released in the environment, enhancing *C. albicans* recognition by the immune system (Gazi et al., 2011). TLR4, which recognizes *O*-linked mannan, works alongside MR to release TNFα, IL-6, IL-10, and IFN-γ (Netea et al., 2006). Dectin-2 and -3 form heterodimers recognizing α-mannans and are involved in secretion of TNFα in RAW264.7 macrophages (Zhu et al., 2013). Furthermore, knockout mice of either of these receptors display a higher mortality rate following intravenous infection as well as an increase in kidney fungal burden (Zhu et al., 2013; Ifrim et al., 2016).

*In vitro* experiments revealed various interactions between fungal molecules and mammalian receptors, reflecting the co-evolution between these organisms. As observed in mice, *C. albicans* recognition triggers immune cell recruitment, which, in the process of killing the yeast, also produces environmental cues that *C. albicans* senses to mask cell surface PAMPs in order to enhance its survival. Additionally, *in vitro* experiments suggest that acidic environments induce glucan exposure, which could be the source for the highly proinflammatory, non-protective innate immune response observed during vulvovaginal candidiasis (Yano et al., 2018).

## Conclusion

The dynamism and diversity exhibited by *C. albicans* in response to environmental cues impact not only its shape and structure but also its interaction with immune cells and responses mounted against the yeast. This protean behavior might explain the successful colonization by *C. albicans* of so many different human niches, but also provides few options for this commensal organism to become pathogenic. While several morphogenesis pathways and virulence factors have been identified, the exact processes governing *C. albicans* masking and unmasking of PAMPs in response to environmental signals require deeper characterization. This knowledge could help develop alternative or complementing strategies to cure *C. albicans* infection at a time where antifungal-resistant strains are becoming a concern.

## Author Contributions

FC wrote the initial draft of the manuscript. RH finalized the manuscript.

### Conflict of Interest

The authors declare that the research was conducted in the absence of any commercial or financial relationships that could be construed as a potential conflict of interest.
